# Acceptance of the COVID-19 Vaccine by Foreigners in South Korea

**DOI:** 10.3390/ijerph182212035

**Published:** 2021-11-16

**Authors:** Chiara Achangwa, Tae-Jun Lee, Moo-Sik Lee

**Affiliations:** 1 Department of Public Health, Graduate School, Konyang University, Daejeon 35365, Korea; ciaraacha@gmail.com (C.A.); leetj1988@naver.com (T.-J.L.); 2 Department of Preventive Medicine, College of Medicine, Konyang University, Daejeon 35365, Korea

**Keywords:** COVID-19 vaccine, vaccine acceptance, vaccine hesitancy, associated factors, foreigners, South Korea

## Abstract

Vaccination against COVID-19 is an important strategy for its control. Assessing the willingness to accept the COVID-19 vaccine in different subgroups is important for an inclusive vaccination program design. Our aim was to determine the COVID-19 vaccine acceptance rate and associated factors among foreigners in South Korea. An online cross-sectional study was carried out from May to June 2021. In this study, 710 individuals participated and most were aged between 26 and 29 (36.1%) years. Overall, 70.8% were willing to receive the vaccine. Males were less likely to accept the vaccine than females (OR: 0.5; 95% CI: 0.4–0.7, *p* < 0.001). Single people were more likely to receive the vaccine than those who were married (OR: 1.4; 95% CI: 0.9–2.0, *p* = 0.04). Other factors associated with willingness to accept COVID-19 vaccine were; vaccine convenience (OR: 1.7; 95% CI: 1.2–2.3, *p* = 0.002), doctors’ recommendation (OR: 2.8; 95% CI: 2.0–3.9, *p* < 0.001), vaccine price (OR: 1.7; 95% CI: 1.2–2.3, *p* = 0.003), vaccine effectiveness (OR: 8.3; 95% CI: 5.8–12.1, *p* < 0.001), vaccine importance (OR: 7.9; 95% CI: 4.6–14.1, *p* < 0.001), and vaccine safety (OR: 6.9; 95% CI: 4.5–10.8, *p* < 0.001). Providing more information on vaccine safety and effectiveness is required to increase vaccine acceptance.

## 1. Introduction

Since the first COVID-19 case in South Korea, the disease has continuously caused great burdens of morbidity and mortality while disrupting economic and social activities [1]. In 2020, the lack of an approved, effective, and safe COVID-19 vaccine made South Korea and other countries consider other non-pharmaceutical interventions to prevent the spread of COVID-19. These interventions included lockdowns, social distancing measures, wearing facemasks, travel restrictions, and quarantine measures [2].

In early 2021, different COVID-19 candidate vaccines were approved and made available to different countries as part of the public health control strategy against COVID-19 [3]. By the end of February 2021, South Korea started COVID-19 vaccination, and priority was given first to those exposed (frontline and medical personnel) and those at risk of high morbidity and mortality (the elderly). According to available data, as of 26 July 2021 (the time of writing of this manuscript), 14.1% of the Korean resident population had been fully vaccinated [4], which was almost three times lower than the percentage of vaccinated individuals in China (40%, as of 28 July 2021) [5]. In order to accelerate vaccination against COVID-19 in the total population, an in-depth understanding of individual willingness to accept the COVID-19 vaccine and associated factors in different subgroups of the population is vital. For effective COVID-19 control using the vaccine, a greater percentage of all residents (Koreans and non-Koreans) should be vaccinated [6]. However, there are several concerns and rumors around the efficacy and safety of the newly developed COVID-19 vaccines [7,8]. Additionally, based on information provided by each country around the globe, nationals have different perceptions and cultural beliefs with respect to receiving the COVID-19 vaccine [9,10].

The initiation of vaccination in South Korea brought up discussions on vaccine acceptance and hesitancy, as the success of the program was dependent on the proportion of vaccine uptake among the entire population. According to the WHO, vaccine hesitancy was one of the top 10 threats to global health in 2019 [11]. Vaccine hesitancy is defined as the delay in acceptance, reluctance, or refusal of vaccination despite the availability of vaccination services [12]. The intention to be vaccinated against the COVID-19 vaccine is recognized as a major issue affecting vaccination programs in different countries.

Empirical literature shows that many studies about COVID-19 vaccine acceptance were carried out between March and December 2020 in many countries [13,14,15]. However, these studies were carried out before the COVID-19 vaccine was approved and made available for use. Authors used hypothetical scenarios and questions to determine the vaccine acceptance or hesitancy in the specific contexts, and the heightened pandemic situation at that time could have had a great influence on participants’ response, affecting the study results [16,17,18,19].

In recent years, due to globalization, South Korea has had an influx of immigrants, primarily students, skilled and unskilled laborers, and marriage-based immigrants, with the number of refugees and asylum seekers steadily increasing [20]. The number of foreigners in South Korea is reported to have greatly increased to approximately 1.3 million legal individuals [21]. These foreigners come from all continents with variations in cultures and languages different from that of South Korea. In particular, nationals from China, Vietnam, Thailand, the USA, and Japan account for the majority of foreigners in Korea [17]. Empirical evidence shows that foreigners generally have difficulties in adequately accessing health care services due to language and cultural differences, which could affect their vaccination intention [20,22]. Additionally, immigrants and even some foreigners have been blamed for the spread of COVID-19 in different countries [23]. These foreigners, regardless of their country of origin, usually belong to speakers of English, Chinese, and a few other foreign languages groups. These groups form strong networks, which aid in the dissemination of information among them, and most often one’s actions or views might likely affect another’s, leading to an indirect collective response to prevailing issues. We also found no prior study on the willingness to accept the COVID-19 vaccine among foreigners in South Korea, which served as a motivation for this study, which happens to be the first study evaluating foreigners’ willingness to accept the COVID-19 vaccine in South Korea.

With ongoing vaccination in South Korea, it is important to have an understanding of the determinants of vaccine acceptance among different population subgroups to ensure appropriate action as well as inclusiveness, which will help achieve sufficient vaccination coverage.

The aim of this study was to determine vaccine acceptance and hesitancy rates and identify factors associated with COVID-19 acceptance among foreigners in South Korea.

## 2. Materials and Methods

### 2.1. Study Design and Setting

Because of the difficulty of conducting face-to-face interviews during the pandemic, an online cross-sectional study was conducted using a web-based questionnaire. Invitations were sent out to participate in this study, which was hosted in Google Forms. This study was carried out between the 5th of May and 20th of June 2021.

### 2.2. Study Participants and Sample Size

The target population included the adult (>18 years of age) foreign population of South Korea. The samples were recruited from all the regions in Korea, and all adults who were able to read as well as understand basic English and had lived for at least 6 months in Korea were considered eligible for participation. The snowball sampling methodology [24] was used to send the survey questionnaire to potential participants. Potential participants were identified by contacting foreign resident associations and platform leaders in Korea, and the survey link, including the inclusion criteria, was publicly disseminated on the social media platforms (E-mail, WhatsApp, Kakao Talk, and others) of these various foreigner groups. Recipients of the study questionnaire were then requested to disseminate it further within their networks of foreigners. All participants gave their consent by signing the informed consent form prior to completing the survey questions. Because there was no previously published data on willingness to accept the COVID-19 vaccine in Korea at the time this study was carried out, the sample size calculation was based on the assumption that the probability of accepting the COVID-19 vaccine was 50% [25], at a confidence interval of 95%, with a precision of 5%. The calculated sample size was 670. By the end of the 3-week time limit to respond to the survey, 710 individuals participated in the study, which exceeded the estimated sample size.

### 2.3. Survey Variables

In order to address the study’s research questions, a set of questions was structured and developed. The survey questionnaire included sections on sociodemographic data, exposure to COVID-19 information, experience with other vaccines, and willingness to accept or reject the COVID-19 vaccine. The survey questionnaire was pre-tested, and after feedback from participants, the questionnaire was revised and finalized based on the feedback.

Participants’ sociodemographic variables were first collected and included; age, gender, marital status, current occupation, level of educational attainment, religion, monthly income (in Korean Won), country, and the continent of origin. Age was grouped into four categories (years old) <25, 26–29, 30–34, and ≥35; educational attainment was grouped into Undergraduate (bachelor degree holders and below) and postgraduate (individuals with masters, Ph.D., and above); current occupation was grouped into student and employed. Because many foreigners were students and on scholarship with a monthly income of 0–2 million won, monthly income (in Korean Won) was grouped into 1–2 million, >2 million, and <1 million won. Marital status was grouped into single and married, and religion was grouped into Christian and Others (Islam, Buddhism, Hinduism, and others). Study participants were also asked whether they had heard about the COVID-19 vaccine and its availability in South Korea or not. In addition, participants were asked whether they were working as a healthcare worker (HCW) or not.

In order to assess the acceptance of the COVID-19 vaccine among foreigners in Korea, the respondents were asked to respond to the question of whether they intend to be vaccinated with the new COVID-19 vaccine using the responses “willing” or “not willing”. If respondents responded with “not willing”, they were asked to provide the reason for their refusal to take the COVID-19 vaccine. To determine factors that could affect participant’s willingness to accept or reject the COVID-19 vaccine, questions on possible factors based on review of relevant literature were asked. Participants were asked if vaccine price, doctor’s recommendation, vaccine convenience, and employer’s recommendation were important in their vaccination decision process. Furthermore, questions were asked to determine respondent’s perception with respect to COVID-19 vaccine safety, effectiveness, and importance. The questionnaire was estimated to take 7 min on average for each participant.

### 2.4. Statistical Analysis

Entries from the survey were recorded in Google Sheets, cleaned, and exported to a local installation of R version 4.0.3 for analysis [26]. Data analysis started by doing descriptive statistics for all variables. Numerical variables were summarized as a mean (±SD), and categorical variables were summarized by frequency and percentage. A bivariate analysis was done between willingness to accept the COVID-19 vaccine (primary outcome variable), and demographic variables, and Chi-square tests were used to assess whether proportions of individuals willing to accept the COVID-19 vaccine differed across categories of demographic variables. By so doing, we compared participants who were willing to be vaccinated to those who were not willing on each potential factor using chi-square tests. Logistic regression models with a binomial distribution family were used to evaluate factors associated with knowledge of the COVID-19 vaccine and its availability in Korea, willingness to accept the COVID-19 vaccine, and the relationship between past vaccine experience and participant’s willingness to accept the COVID-19 vaccine. The level of statistical significance was set at a *p*-value < 0.05, and all statistical tests were two-tailed.

## 3. Result

### 3.1. Demographic Distribution of Study Participants and Knowledge of COVID-19 Vaccine Availability in Korea

In total, 710 foreigners, 60.7% of whom were males, completed the study questionnaire. In this study, 81.4% of the participants reported having knowledge of the availability of the COVID-19 vaccine in South Korea. The majority of participants were aged between 26 and 29 years (36.1%), and 55.2% of the participants were Asians. Most study participants were post-graduates (71.1%). Non-healthcare workers accounted for 87.2% of the total participants. Individuals in the age group 26–29 years (OR: 4.5; CI: 2.6–7.8, *p* < 0.001) were more likely to have knowledge about the COVID-19 vaccine availability than individuals in the other age groups. Males (OR: 0.7; CI: 0.4–1.0, *p* = 0.048), undergraduates (OR: 0.6; CI: 0.4–0.9, *p* = 0.021), and singles (OR: 0.5; CI: 0.3–0.7, *p* = 0.001) were less likely to have knowledge about the COVID-19 vaccine availability than their counterparts. (Table 1).

### 3.2. Acceptance of COVID-19 Vaccine by Foreigners in South Korea

Overall, 70.8% of the study participants reported willingness to receive the new COVID-19 vaccine while 29.2% were hesitant. There was a significant difference between foreigners who were willing to receive the COVID-19 vaccine and those not willing to receive the vaccine by gender (*p* < 0.001), level of education (*p* = 0.04), region of origin (*p* < 0.001), and religion (*p* < 0.001) (Table 2). 

### 3.3. Reason for Not Willing to Receive the COVID-19 Vaccine

Among those who were not willing to receive the COVID-19 vaccine, the reasons for unwillingness were as follows: 70.5% were concerned about the side effects of the vaccine, 13.4% were concerned about the vaccine safety, 13.8% reported that they feared the vaccine was not effective, and 2.3% reported other reasons including the lack of confidence in the government policies about the COVID-19 vaccine.

### 3.4. Relationship between Past Vaccine Experience and COVID-19 Vaccine Intention

A significant difference between past vaccine experience and intention to accept COVID-19 vaccine was observed, with those who had not refused any vaccine in the past more likely to accept the COVID-19 vaccine compared to those who had refused any vaccine in the past (OR: 0.5, 95% CI: 0.3–0.7, *p* < 0.001).

### 3.5. Associated Factors of Foreigners’ Willingness to Accept COVID-19 Using Logistic Regression Analysis

Table 3 presents the logistic regression analysis for factors associated with foreigners’ willingness to accept the COVID-19 vaccine. In our logistic regression model, males were less likely to accept the vaccine than females (OR: 0.5; 95% CI: 0.4–0.7, *p* < 0.001). Participants who were single were 1.4 times more likely to receive the vaccine than those who were married (OR: 1.4; 95% CI: 1.0–2.0, *p* = 0.04). Undergraduates were more likely to receive the vaccine than post-graduates (OR: 1.5; 95% CI: 1.0–2.2, *p* = 0.033). Other factors that were associated with willingness to accept COVID-19 vaccine were vaccine convenience (OR: 1.7; 95% CI: 1.2–2.3, *p* = 0.002), doctors’ recommendation (OR: 2.8; 95% CI: 2.0–3.9, *p* < 0.001), vaccine price (OR: 1.7; 95% CI: 1.2–2.3, *p* = 0.003), vaccine effectiveness (OR: 8.3; 95% CI: 5.8–12.1, *p* < 0.001), vaccine importance (OR: 7.9; 95% CI: 4.6–14.1, *p* < 0.001), and vaccine safety (OR: 6.9; 95% CI: 4.5–10.8, *p* < 0.001) (Table 3).

## 4. Discussion

Vaccination is believed to be the best method for the prevention of diseases. However, its acceptance differs with various factors including time, human behavior, social class, and ethnicity [26,27,28,29]. Our study outlines vaccine acceptance and hesitancy rate and identifies factors associated with COVID-19 acceptance among foreigners in South Korea.

Overall, 81.41% of all respondents had knowledge about the COVID-19 vaccine and its availability in South Korea. This result was encouraging and indicative that messages about the COVID-19 vaccine disseminated by health authorities were being received by the foreign resident population. Studies have shown that differences in knowledge about COVID-19 vaccine availability could also affect willingness to accept the COVID-19 vaccine [30]. We found that knowledge about the COVID-19 vaccine and its availability in Korea was associated with different factors. Compared to other age groups, individuals in the age group of 26–29 years were more likely to have knowledge about the COVID-19 vaccine and its availability. We believe that members of this age group are more likely to be engaged in different activities on social media, which was used by many international organizations as well as health ministries to disseminate information about COVID-19. Students compared to employed individuals were less likely to have knowledge about the COVID-19 vaccine and its availability. This could be explained by the fact that most employers required that their employees be vaccinated immediately when the vaccine was available and opened to the public. These employers constantly provided their employees with COVID-19 vaccine availability information.

Singles compared to married individuals were less likely to have knowledge about the COVID-19 vaccine and its availability. Married individuals are most likely to worry about the safety and welfare of their families, so they may be constantly searching for information about the vaccine and its availability.

Compared to undergraduate students, postgraduate students were more likely to have knowledge about the COVID-19 vaccine availability. Postgraduate education is mostly made of discussions, research, and interactions at various levels, which opens up these students much more to different sources of information.

Additionally, males were less likely to have knowledge about the COVID-19 vaccine and its availability compared to females. Literature shows that women are more likely to be involved with preventive behaviors than men [31], which could explain their getting more information about the COVID-19 vaccine availability.

We found that 70.8% of foreign residents in South Korea are willing to receive the COVID-19 vaccine. Despite all the controversies and misinformation about COVID-19, this indicates a positive attitude toward COVID-19 vaccination among the foreign resident population with a high demand for the vaccine and the high recognition of the importance of vaccines in the fight against COVID-19. These results were lower than those of a study by Wang et al. in China with a vaccine acceptance rate of 91.3% [13] but higher than a study by Wong et al. [14] with a vaccine acceptance rate of 37.2% and Shekhar et al. with a 36% vaccine acceptance rate [32]. These differences could be due to the timing of studies, data collection, and the way questions were asked across the studies. Contrary to other studies that were carried out before the availability of an approved COVID-19 vaccine, our study was carried during the first few months of COVID vaccine availability. Our finding is one of the first estimates of acceptability of the COVID-19 vaccine in South Korea and can be used to guide interventions with respect to COVID-19 vaccine uptake. However, temporal changes may be observed since willingness to accept may not always result in the actual accepting behavior [17].

By sociodemographic characteristics, the percentage of female respondents who were willing to accept the COVID-19 vaccine was higher than for males (78.9% vs. 65.7%). Our results are similar to those from Wong et al., who found that females had a 71.2% vaccine acceptance rate compared to 28.2% males [14]. We attributed this to the fact that females were more likely to engage in preventive behavior than males. However, some studies show that females are less likely to believe that the vaccine will protect the health of those who are vaccinated and are less willing to take the vaccine than males [33,34]. This lack of conviction by women has been reported by other studies [33,34]. A study carried out in eight OECD countries focusing on a detailed analysis regarding gender-based issues pertaining to the pandemic demonstrated similar results and recommended gender-based public health policies and communication [31].

Additionally, those from the Asian region had a higher vaccine acceptance rate (56.9%) probably due to well-organized government campaigns and population awareness about COVID-19 vaccination. However, it is encouraging that the COVID-19 vaccine acceptability was high among most of the participants. Health education and promotion activities targeted at subgroups of foreigners with a lower vaccine acceptancy rate may help increase the rate of vaccine uptake.

In this study, 29.2% of participants were not willing to receive the vaccine. Additionally, in this group, 70.51% were concerned about the side effects of the vaccine, 13.39% were concerned about the vaccine safety, and 13.84% reported that they feared the vaccine was not effective. These concerns about vaccine safety, effectiveness, and side effects have frequently been reported as major obstacles to vaccination decision-making, specifically for newly introduced vaccines, which have not been fully tested [35]. Recently, some studies have reported that fully vaccinated individuals against COVID-19 have been infected with the COVID-19 virus [36]. This could further increase the vaccine hesitancy rate if not well managed. In response to this, we urge the health departments to consider rigorous education programs for COVID-19 in order to improve overall vaccine confidence and vaccine compliance among all groups.

Another important finding of this study was on past vaccine experience and intention to accept the COVID-19 vaccine. Individuals who had not refused any vaccine in the past were more likely to accept the COVID-19 vaccine. Authors think that individuals who have a history of refusing vaccines would likely refuse the COVID-19 vaccine regardless of information provided, and this could be the main reason they would not accept the vaccine. This pattern of behavior of vaccine refusal has been noted in some individuals [37]. This is in line with past studies which showed that individuals with no past hesitation in receiving a vaccine are more likely to be vaccinated and will recommend vaccination to friends and family [38].

Finally, different factors were associated with the willingness to accept the COVID-19 vaccine. These factors include gender, level of education, region of origin, doctor’s recommendation, past vaccine experience, vaccine convenience, effectiveness, importance, and safety. We noted that willingness to accept the COVID-19 vaccine was positively associated with doctor’s recommendation, past vaccine experience, vaccine convenience, effectiveness, importance, and safety. The authors believe that due to the evident uncertainties surrounding COVID-19 vaccine development, doubts have been instilled in individuals with respect to vaccine convenience, effectiveness, importance, and safety, which could be a reason for their association with the willingness to accept the vaccine. The speed of the vaccine development as well as concerns about its side effects [37] could also be reasons for these observations. Doctors, as well as health care workers are generally trusted by the population on health-related issues. This explains the fact that doctors’ recommendation of the COVID-19 vaccine was positively associated with willingness to accept the COVID-19 vaccine. These results were similar to the study by Earnshaw et al., who reported that doctors were the most trusted source of information about COVID-19, and doctors’ recommendation was strongly correlated to vaccine acceptance [39].

The major strengths of our study included the diverse representation from different genders, age groups, and cultural backgrounds. However, our study had some limitations. This study was an online study and relied on self-reported behaviors, which may be affected by recall bias. Additionally, social desirability bias could also affect the interpretation of our study results, though the responses were anonymized to minimize this. Because our study was a cross-sectional study, we were unable to establish cause-and-effect relationships.

## 5. Conclusions

Globally, there is evidence of vaccine hesitancy among different population subgroups, including nationals and foreigners, for different reasons. However, there is a lack of comprehensive literature investigating this aspect in these different population subgroups in South Korea. To date, no study was found documenting the COVID-19 vaccine acceptance rate among foreigners in Korea. To bridge this gap, we investigated the vaccine acceptance and hesitancy rates and identified the factors associated with COVID-19 acceptance among foreigners in South Korea through an online cross-sectional study. Our results showed that 70.8% of foreign residents in South Korea reported willingness to accept the COVID-19 vaccine, while 29.2% were not willing. In addition, vaccine acceptance was associated with many factors such as gender, level of education, region of origin, marital status, vaccine convenience, doctors’ recommendation, vaccine price, vaccine effectiveness, vaccine importance, and vaccine safety.

A key limitation within our study was its online nature, which relied on self-reported behaviors, with a probability of recall bias. Despite its limitations, this study sheds light on the vaccine acceptance rate and associated factors related to the COVID-19 vaccine among foreigners. It also demonstrates the need to enhance knowledge about the COVID-19 vaccine and to eliminate unfounded theories with scientifically proven facts. Concerns about vaccine convenience, effectiveness, importance, and safety may hinder the COVID-19 vaccine acceptance rate in this group. Intensive health education on the COVID-19 vaccine from reliable sources is required to increase the trust and willingness to accept the vaccine in this group.

Future research in this area should focus on finding out what proportion of this subgroup actually received the vaccine and what proportion did not receive it in comparison with nationals for a better understanding of attitudes, perceptions, and behavioral patterns. In addition, future research may also provide further insights on how willingness to accept the COVID-19 vaccine changes over time in different population subgroups and identify factors associated with such changes.

## Figures and Tables

**Table 1:** Sociodemographic characteristics of study participants by their knowledge about COVID-19 vaccine and its availability in South Korea (*N* = 710).

Variable	Knowledge of COVID-19 Vaccine and Its Availability in Korea				
	Total, *n* (%)	I Don’t Know, *n* (%)	No, *n* (%)	Yes, *n* (%)	* OR (95% CI) [*p*-Value]
**Age/Years**					
<25	118 (16.6)	18 (15.3)	24 (20.3)	76 (64.4)	1 [Reference]
26–29	256 (36.1)	9 (3.5)	19 (7.4)	228 (76.4)	4.5 (2.6–7.8) [<0.001]
30–34	209 (29.4)	12 (5.7)	20 (9.6)	177 (84.7)	3.1 (1.8–5.2) [<0.001]
≥35	127 (17.9)	17 (13.4)	13 (10.2)	97 (76.4)	1.8 (1.0–3.1) [0.041]
**Gender**					
Female	279 (39.3)	17 (6.1)	25 (9.0)	237 (84.9)	1 [Reference]
Male	431 (60.7)	39 (9.1)	51 (11.8)	341 (79.1)	0.7 (0.4–1.0) [0.048]
**Level of Education**					
Postgraduate	205 (28.9)	37 (7.3)	46 (9.1)	422 (83.6)	1 [Reference]
Undergraduate	505 (71.1)	19 (9.3)	30 (14.6)	156 (76.1)	0.6 (0.4–0.9) [0.021]
**Income/KRW**					
<1 million won	308 (43.4)	38 (12.3)	33 (10.7)	237 (76.9)	1 [Reference]
>2 million won	171 (24.1)	6 (3.5)	15 (8.8)	150 (87.7)	2.1(1.3–3.7) [0.005]
1–2 million won	205 (28.9)	10 (4.9)	24 (11.7)	171 (83.4)	1.5 (1.0–2.4) [0.077]
**Region**					
Africa	188 (26.5)	14 (7.5)	27 (14.4)	147 (78.2)	1 [Reference]
Asia	392 (55.2)	33 (8.4)	37 (9.4)	322 (82.1)	1.3 (0.8–1.9) [0.258]
Others	130 (18.3)	9 (6.9)	12 (9.2)	109 (83.8)	1.5 (0.8–2.6) [0.212]
**Marital Status**					
Married	230 (32.4)	15 (6.5)	11 (4.8)	204 (88.7)	1 [Reference]
Single	480 (67.6)	41 (8.5)	65 (13.5)	374 (77.9)	0.5 (0.3–0.7) [0.001]
**Occupation**					
Employed	201 (28.3)	11 (5.5)	12 (6.0)	178 (88.6)	1 [Reference]
Student	508 (71.5)	45 (8.9)	64 (12.6)	399 (78.5)	0.5 (0.3–0.8) [0.002]
**Religion**					
Christians	427 (60.1)	34 (7.9)	52 (12.2)	341 (79.9)	1 [Reference]
Others	283 (39.9)	22 (7.8)	24 (8.5)	237 (83.7)	1.3 (0.9–1.9) [0.193]
**Health Care Related Job**					
No	619 (87.2)	53 (8.6)	68 (11)	498 (80.5)	1 [Reference]
Yes	91 (12.8)	3 (3.3)	8 (8.8)	80 (87.9)	1.8 (0.9–3.6) [0.091]

*n*: number; * we used logistic regression model to evaluate the factors associated with having knowledge of COVID-19 vaccine and its availability in Korea; level of significance set at *p*-value < 0.05.

**Table 2:** Bivariate associations between sociodemographic characteristics and willingness to take the COVID-vaccine (*N* = 710).

	Acceptance of COVID-19 Vaccine			
Variable	Total, *n* (%)	Not Willing, *n* (%)	Willing, *n* (%)	*p*-Value
**Age**				0.444
<25	118 (16.6)	39 (33.1)	79 (66.9)	
26–29	256 (36.1)	69 (26.9)	187 (73.1)	
30–34	209 (29.4)	66 (31.6)	143 (68.4)	
≥35	127 (17.9)	33 (26.0)	94 (74.0)	
**Gender**				<0.001
Female	279 (39.3)	59 (21.1)	220 (78.9)	
Male	431 (60.7)	148 (34.3)	283 (65.7)	
**Level of Education**				0.04
Postgraduate	505 (71.1)	159 (35.5)	346 (68.5)	
Undergraduate	205 (28.9)	48 (23.4)	157 (76.6)	
**Income**				0.507
<1 million won	308 (43.4)	95 (30.8)	213 (69.2)	
>2 million won	171 (24.1)	45 (26.3)	126 (73.7)	
1–2 million won	205 (28.9)	56 (27.3)	149 (72.7)	
**Region**				<0.001
Africa	188 (26.5)	85 (45.2)	103 (54.8)	
Asia	392 (55.2)	106 (27.0)	286 (73.0)	
Others	130 (18.3)	16 (12.3)	114 (87.7)	
**Marital Status**				0.065
Married	230 (32.4)	78 (33.9)	152 (66.1)	
Single	480 (67.6)	129 (26.9)	351 (73.1)	
**Current Occupation**				0.092
Employed	201 (28.3)	49 (24.4)	152 (75.6)	
Student	508 (71.5)	158 (31.1)	350 (68.9)	
**Religion**				<0.001
Christian	427 (60.1)	166 (38.9)	261 (61.1)	
Others	283 (39.9)	41 (14.5)	242 (85.5)	
**Health-Care Related Job**				0.994
No	619 (87.2)	181 (29.2)	438 (70.8)	
Yes	91 (12.8)	26 (28.6)	65 (71.4)	

*n*: number; *p*-value: Chi-squared test was used to determine the differences between categorical variables; level of significance set at *p*-value < 0.05.

**Table 3:** Logistic regression analysis for factors associated with willingness to accept COVID-19 vaccine among foreigners in South Korea (*N* = 710).

Factor	OR (95% CI) [*p*-Value]
**Age**	
<25	1 [Reference]
26–29	1.3 (0.8–2.1) [0.227]
30–34	1.1 (0.7–1.7) [0.784]
≥35	1.4 (0.8–2.5) [0.226]
**Gender**	
Female	1 [Reference]
Male	0.5 (0.4–0.7) [<0.001]
**Level of Education**	
Postgraduate	1 [Reference]
Undergraduate	1.5 (1.0–2.2) [0.033]
**Income**	
<1 million won	1 [Reference]
>2 million won	1.3 (0.8–1.9) [0.297]
1–2 million won	1.2 (0.8–1.8) [0.391]
**Region**	
Africa	1 [Reference]
Asia	2.2 (1.6–3.2) [<0.001]
Others	5.9 (3.3–11) [<0.001]
**Current Occupation**	
Employed	1 [Reference]
Student	0.7 (0.5–1.0) [0.077]
**Marital Status**	
Married	1 [Reference]
Single	1.4 (1–2) [0.04]
**Religion**	
Christian	1 [Reference]
Others	3.8 (2.6–5.6) [<0.001]
**Health-Care Related Job**	
No	1 [Reference]
Yes	1.0 (0.6–1.7) [0.896]
**Vaccine Convenience**	
No	1 [Reference]
Yes	1.7 (1.2–2.3) [0.002]
**Doctor’s Recommendation**	
No	1 [Reference]
Yes	2.8 (2.0–3.9) [<0.001]
**Vaccine Price**	
No	1 [Reference]
Yes	1.67 (1.2–2.3) [0.003]
**Vaccine Effectiveness**	
No	1 [Reference]
Yes	8.33 (5.8–12.1) [<0.001]
**Refused Any Vaccine in the Past**	
No	1 [Reference]
Yes	0.52 (0.4–0.8) [<0.001]
**Vaccine is Important**	
No	1 [Reference]
Yes	7.9 (4.6–14.1) [<0.001]
**Vaccine Safety**	
I don’t know	1 [Reference]
No	0.328 (0.2–0.5) [<0.001]
Yes	6.93 (4.5–10.8) [<0.001]

COVID-19: Coronavirus disease 2019; OR: Odds Ratio; CI: Confidence interval; level of significance set at *p*-value < 0.05.

## Data Availability

Data will be made available upon request. Please contact the corresponding author of this article and data will be provided to you within 24 h. The data are not publicly available due to privacy reasons.
